# The Mother-Infant Study Cohort (MISC): Methodology, challenges, and baseline characteristics

**DOI:** 10.1371/journal.pone.0198278

**Published:** 2018-05-31

**Authors:** Hadia Radwan, Mona Hashim, Reyad Shaker Obaid, Hayder Hasan, Farah Naja, Hessa Al Ghazal, Hamid Jan Jan Mohamed, Rana Rizk, Marwa Al Hilali, Rana Rayess, Ghamra Izzaldin

**Affiliations:** 1 Department of Clinical Nutrition and Dietetics, College of Health Sciences, Research Institute of Medical and Health Sciences(RIMHS), University of Sharjah, Sharjah, United Arab Emirates; 2 Department of Nutrition and Food Sciences, American University of Beirut, Beirut, Lebanon; 3 Family Health Promotion Center, Sharjah, United Arab Emirates; 4 Nutrition and Dietetics Program, Universiti Sains Malaysia, Kelantan, Malaysia; 5 Department of Health Services Research, Maastricht University, Maastricht, The Netherlands; 6 Institut National de Santé Publique, d’Épidémiologie Clinique et de Toxicologie (INSPECT-LB), The Lebanese University, Beirut, Lebanon; McMaster University, CANADA

## Abstract

**Background:**

The United Arab Emirates (UAE) exhibits alarming high prevalence of Non-Communicable Diseases (NCDs) and their risk factors. Emerging evidence highlighted the role of maternal and early child nutrition in preventing later-onset NCDs. The objectives of this article are to describe the design and methodology of the first Mother and Infant Study Cohort (MISC) in UAE; present the baseline demographic characteristics of the study participants; and discuss the challenges of the cohort and their respective responding strategies.

**Methods:**

The MISC is an ongoing two-year prospective cohort study which recruited Arab pregnant women in their third trimester from prenatal clinics in Dubai, Sharjah and Ajman. Participants will be interviewed six times (once during pregnancy, at delivery, and at 2, 6, 12 and 24months postpartum). Perinatal information is obtained from hospital records. Collected data include socio-demographic characteristics, lifestyle, dietary intake and anthropometry; infant feeding practices, cognitive development; along with maternal and infant blood profile and breast milk profile.

**Results:**

The preliminary results reported that 256 completed baseline assessment (mean age: 30.5±6.0 years; 76.6% multiparous; about 60% were either overweight or obese before pregnancy). The prevalence of gestational diabetes was 19.2%. Upon delivery, 208 women-infant pairs were retained (mean gestational age: 38.5±1.5 weeks; 33.3% caesarean section delivery; 5.3% low birthweight; 5.7% macrosomic deliveries). Besides participant retention, the main encountered challenges pertained to cultural complexity, underestimation the necessary start-up time, staff, and costs, and biochemical data collection.

**Conclusions:**

Despite numerous methodological, logistical and sociocultural challenges, satisfactory follow-up rates are recorded. Strategies addressing challenges are documented, providing information for planning and implementing future birth cohort studies locally and internationally.

## Introduction

Adequate nutrition during “the first 1000 days of life”, i.e. the period extending from conception up to the child’s second year of life, greatly affects fetal growth and birth outcomes, and is essential to ensure health and development of infants and children to their full potential [1–6]. Proper breastfeeding and complimentary feeding practices during this period have a lasting impact on a child’s health [7,8], and can save the life of 800,000 infants each [9]. Consequently, the World Health Organization (WHO) and the United Nations Children’s Fund (UNICEF) recommend that infants be exclusively breastfed for the first six months of life, up to two years of age or beyond [7,10,11].

Furthermore, early-life exposures that influence growth during development, including genetic, epigenetic, nutritional and environmental, may modulate adult disease risk [12]. Recent scientific literature is investigating the period extending from gestation to infancy and early childhood as a window of opportunity for curbing the non-communicable disease (NCD) pandemic through developmental programming [13,14]. In specific, high pre-pregnancy weight and weight gain and mother’s diet during early gestation were associated with an adverse cardio-metabolic profile in the offspring, such as increased risk of childhood body mass index (BMI), body fat and systolic blood pressure [13,15]. Understanding the nature of these exposures might be a key to preventing disease and improving health [16].

Population-based prospective cohort studies are key study designs for investigating life course processes [17]. Pregnancy and birth cohort studies are especially suited for exploring the origins of health and disease that begin as early as in pre-pregnancy, fetal life and infancy. The strength and liability of these longitudinal studies lie in their potential to explore causal relationships whilst requiring less recall than other epidemiological study designs [18,19]. Therefore, well-designed and well-conducted birth cohort studies are particularly valuable for understanding the role of pre- and postnatal environment on immediate and future health [12].

Until recently, conducting cohorts addressing maternal and child nutrition and its impact on health outcomes in developing countries was limited [20–22], especially in the Middle East region [23–25]. Generating birth cohort data from this region is useful for exploring country- and population-specific early-life predictors of health and disease. This is essential especially for countries where the economic transition and subsequent unplanned lifestyle and nutrition transition has had devastating consequences on the population health [26,27]. In particular, the United Arab Emirates (UAE) exhibited in recent years an enormous economic growth, that was paralleled with surging rates of NCDs and their risk factors in adult and pediatric populations [26,27] as well as a dramatically growing probability of premature death [28]. Changes in infant feeding practices were not isolated from the nutrition transition in the country; rather, a decline in exclusive breastfeeding, a high dependence on bottle-feeding and suboptimal weaning practices are being increasingly reported [29,30]. Taking into consideration the resulting high morbi-mortality and economic losses, designing and implementing appropriate NCD prevention and control programs through translational research are at the top of local and regional agendas [31,32]. To move these agendas forward, the Mother-Infant Study Cohort (MISC) is launched. The MISC aims to longitudinally investigate the effects of nutrition and lifestyle characteristics on birth outcomes, infant nutritional status and cognitive development, as well as early-life cues of NCDs, with a focus on the Emirati setting. Also, the findings of the MISC are planned to be used for a cross-country comparison with other Gulf-based cohorts, such as the ongoing Mother and Infant Nutritional Assessment (MINA) cohort study in Qatar and Lebanon [25]. The ultimate aims of the MISC are to advance scientific research on women’s and children’s health in the UAE and Arab region, to act as a model for generating evidence-based findings, translating them into culturally-specific and targeted practice recommendations, and to advocate for a meaningful change at the policy level.

Although, birth cohorts and life course designs offer opportunities, their implementation is uniquely challenging [33,34]. Being the first birth cohort in the UAE, the MISC faced numerous methodological, logistical, and sociocultural challenges. It is important to present an overview of the methodology, protocol and challenges of the MISC to inform the planning and implementation of future birth cohort studies locally, regionally and internationally. Accordingly, the objectives of this article are to: describe the design and methodology of the MISC; present the baseline demographic characteristics of the study participants; and discuss the challenges of the cohort and their respective responding strategies.

## Materials and methods

### Study design and overview

MISC is the first prospective cohort study in the UAE to comprehensively investigate maternal and infant factors in relation to child health outcomes, as well as early-life determinants of NCDs, through integration of sociodemographic, dietary, lifestyle, anthropometric and biological and cognitive data. At present, recruitment of study participants is completed; data collection and preliminary analyses are being conducted.

### Ethical approval

The study was approved by the Research and Ethics Committee, University of Sharjah (REC/14/01/1505) and by Al Qassimi Clinical Research Centre Ethical Research Committee (REC Reference Number: 215 12015–03), by the Ministry of Health Ethical Research Committee (R02) and by Dubai Health Authority (DSREC-0/2016). The research ethics bodies focused on ensuring adherence to the regulatory requirements for the protection of pregnant women and babies as vulnerable research subjects, throughout all study procedures (consent documentation, data collection including blood withdrawal requirements, and data transfer). It was thus requested by Al Qassimi Clinical Research Centre to prepare a separate consent form to obtain the fathers’ approval on the behalf of the infants, since according to Emirati law, the father is the legal guardian. Moreover, special considerations for blood withdrawal by pediatric-certified phlebotomists were noted.

### Study setting and population

Participants were recruited from antenatal clinics in three main public governmental hospitals, and seven Primary Health Care (PHC) clinics and Mother and Child Centers (MCH) in the Emirates of Sharjah, Dubai and Ajman. The research team visited the pre-specified clinics, screened eligibility criteria of pregnant women attending the clinics, informed them of the objectives, conduct and timeline of the study and invited them to participate. The study followed a convenient sampling and did not adopt a special advertisement for recruitment. Eligible women were asked, along with the fathers, to provide signed consent to participate in the study, and were given a copy of it. The participants were chosen according to the following criteria.

**Inclusion criteria**
- Pregnant women:
  - Emirati or Arab expatriate
  - Age: 19–40 years
  - Singleton pregnancy
  - Within the third trimester of pregnancy (27–42 weeks of gestation)
  - Free of chronic diseases (diabetes, hypertension, kidney disease, cancer, or other chronic diseases), autoimmune disorders, or infections with the human immunodeficiency virus, or hepatitis in preconception
  - Receiving antenatal care in any of the above-mentioned clinics and expected to give birth in a participating public hospital
  - Not planning to permanently leave the UAE during the timeline of the study.
- Infants:
  - Early term (37 weeks of gestation) or full term (between 39 weeks 0 days, and 40 weeks 6 days of gestation) live birth infants
  - Free of major illnesses (congenital diseases or any health condition that could deter *breastfeeding*, *such as cleft lip or cleft palate)*, *i*.*e*. *apparently healthy infants*.

**Exclusion criteria**
- Pregnant women:
  - Multiple pregnancy (pregnant with two or more fetuses)
  - High risk pregnancy or pre-eclampsia
  - History of chronic diseases.
- Infants:
  - Preterm (<37 weeks of gestation) or late term (>42 weeks of gestation) infants
  - Congenital diseases or any health condition that could deter breastfeeding.

Bearing in mind that loss to follow-up might be “not at random” and lead to biased estimates [35], different strategies were put place to increase participation and retention in the cohort: sharing the biomedical results with the study participants at 7-month postpartum; providing gift vouchers and toiletries for attendance to each time-point, ensuring transportation coverage, if needed. In an attempt to increase retention, text messages will be sent to each participant at each time-point. Recruitment was completed during June 2016.

### Sample size and power analysis

As mentioned above, one of the main purposes of the MISC is to explore the relationship between maternal characteristics and infant outcomes. When the assessed variables are both continuous, we often resort to Pearson’s correlation coefficient (*r*), enabling the assessment of a linear dependence (correlation) between the variables, as well as the strength and direction of this correlation. The absolute value of *r* ranges between 0 and 1, and its increasing magnitude indicates a stronger association. Taken the limited data on the above-mentioned associations, *r* = |0.2| was chosen, as it yields the largest sample size. An *r*<|0.2| indicates a weak correlation- usually considered too small to be clinically significant. Using the Power Analysis and Sample Size System (PASS) software version 11, the sample size needed to detect a correlation as small as |0.2|, at 80% power and 5% type I error, is determined at 193 (rounded-up at 200). Another way of assessing the relationship between maternal and infant-related factors is through regression analysis, where an outcome variable is regressed on a number of explanatory variables (predictors). Based on the rationale of considering 10–15 observations per predictor, the sample size of 200 would be sufficient to conduct regression analyses on up-to 15 predictors. Since it is not expected to exceed this number of independent variables per regression model, we considered the sample size of 200 as adequate. Considering that previous birth cohorts showed a retention rate of 85% [36], it was anticipated that around 15% of the participants will be lost after the first visit. Furthermore, we expected to lose an additional 10% due to preterm delivery and another 10% due to still birth. As such, the original sample size was inflated by 35% and the target sample size was determined at 270 pregnant women. We expect to retain around 65% of the participants at two years postpartum.

### Study protocol

Assessments for this cohort were planned over six time-points, extending from late pregnancy till two years postpartum. The postpartum time-points were chosen in parallel with the immunization schedule in the UAE, when mothers are expected to visit the PHCs/MCHs to vaccinate their infants. The cohort assessments and timeline are detailed in Table 1. Data were collected via interviewer-administered questionnaires, anthropometric measurements, dietary records, bio-specimens (blood and breast milk) analyses and review of hospital electronic medical records. Data collection tools and methods are detailed below.

**Table 1:** Data collection and study timeline.

Detailed information		Baseline	Delivery	Postpartum (Months)			
		3^rd^ trimester (27–40 weeks)	0–10 days	2	6	12	24
**Inclusion** **Mother**: healthy, 19–40 years old, Arab		✓					
**Infant**: full term, without major illness			✓				
**MATERNAL**	**Sociodemographic characteristics** Age, Place of residence, Nationality, Occupation, Education, Parity, Income	✓					
	**Dietary intake** 24hr recall, Supplement use	✓		✓	✓		
	FFQ	✓		✓			
	**Life style practices** Smoking, Physical activity	✓				✓	
	**Anthropometry**						
	Height	✓					
	Pre-pregnancy weight	✓					
	Weight, Waist circumference, Body fat	✓	✓	✓	✓	✓	✓
	Blood pressure	✓				✓	✓
	**Blood** Lipid profile, Glucose, Insulin, HMW Adiponectin, IL-6, TNF-α, hs-CRP, Vitamin D		✓		✓		
	**Breast milk** Fatty acids, Oligosaccharides, Insulin, HMW Adiponectin, IL-6, TNF-α, hs-CRP (proximate analysis)		✓				
**Perinatal information** Pregnancy complications: GDM		✓					
Delivery method & complications			✓				
**INFANT**	**Sociodemographic characteristics** Gestational age, Birth date, Gender	✓	✓				
	**Anthropometry** Weight, Length, Head circumference	✓	✓	✓	✓	✓	✓
	Mid upper arm circumference			✓	✓	✓	✓
	**Feeding practices** Breastfeeding, Formula & Complimentary			✓	✓	✓	✓
	Eating environment at home					✓	✓
	**Dietary intake** 24hr recall, Supplement intake			✓	✓	✓	✓
	**Blood** Lipid profile, Glucose, Insulin, HMW Adiponectin, IL-6, TNF-α, hs-CRP, Vitamin D				✓		
	**Cognitive evaluation** Ages & stages					✓	✓

FFQ: food frequency questionnaire, HMW Adiponectin: high molecular weight adiponectin; IL-6: interleukin-6; TNF-α: tumor necrosis factor-alpha; *hs*-CRP: high-sensitivity C-reactive protein; GDM: gestational diabetes mellitus.

### Questionnaires

Multi-component questionnaires used for this study were adapted from the MINA cohort study [25]. These are content-validated, culturally-adapted and pilot-tested questionnaires that were revised for use in this study among Emirati pregnant women. The Arabic version of the questionnaires were administered by trained research nutritionists.

**Maternal sociodemographic characteristics and lifestyle**: sociodemographic factors include age, nationality, place of residence, occupation, education, parity, and income. Lifestyle practices include physical activity, cigarette and shisha (narghile/water pipe) smoking. Physical activity was assessed using the Pregnancy Physical Activity Questionnaire (PPAQ) [37] during the third trimester of pregnancy, and the short International Physical Activity Questionnaire (IPAQ) [38] during postpartum. Metabolic equivalents (METs) were computed, and physical activity levels were classified as low, moderate or high-intensity. Maternal cigarette smoking dose will be assessed according to Bachir and Chaaya [39], maternal shisha smoking dose will be assessed according to Tamim et al. [40], These questions were extended with one question inquiring about other smokers living in the same house.

**Maternal dietary intake**: maternal intake was evaluated using a culture-specific food frequency questionnaire (FFQ) and multiple-pass 24-hour dietary recalls. The used FFQ is a semi-quantitative questionnaire consisting of 89 items, grouped into 24 food categories comprehensively representing the Gulf and Middle Eastern cuisine. The 24-hour recalls were assessed using the United States Department of Agriculture’s Multiple Pass Food Recall (MPR), which attenuates recall bias [41,42]. For the FFQs and 24-hour recalls, participants were assisted with the reference portions of the two-dimensional food portion visual (Millen and Morgan, Nutrition Consulting Enterprises, Framingham, Massachusetts, United States), as well as supplementary visual aids about portion sizes of common items in the traditional Gulf and Middle Eastern cuisine meals [43], to help estimating ingested quantities. Daily energy and nutrient intakes will be calculated using the food composition tables provided by the NUTRIONIST PRO^™^ diet analysis software (Axxya Systems LLC., USA, version 5.1.0, 2014, First Fata Bank, Nutritionist Pro, San Bruno, CA) and the food composition table of Middle Eastern foods for local and traditional dishes [44]. Supplement use were assessed via questions on the type, dose and frequency of used vitamins, minerals, herbal or other products.

**Infant feeding practices and dietary intake**: breastfeeding and complementary feeding practices will be assessed based on WHO indicators [45], and categorized as exclusive breastfeeding, predominant breastfeeding, or complementary feeding. In order to assess the infant’s complementary feeding, mothers will be asked about the timing of introduction and type of solid, semi-solid and soft foods. Minimum dietary diversity, minimum meal frequency, minimum acceptable diet, and consumption of iron-rich or iron-fortified foods will be assessed.

**Infant cognitive development**: assessed using the 12 and 24-month Ages and Stages Questionnaire (ASQ). The ASQ system is originally a parent-completed questionnaire but may also be completed by a professional interacting with the infant and the parent. The ASQ is a developmental screening tool, widely used in pediatric populations. It exhibits high measures of sensitivity, specificity, and reliability [46]. Total scores will be computed for communication, gross motor, fine motor, problem solving and personal-social areas, and assessed based on the cut-off values suggested by Squires and Bricker [47]. The Arabic versions provided by the publisher will be used.

### Anthropometry

**Maternal anthropometric assessment**: height, weight, waist circumference and blood pressure were measured following standard techniques. Pre-pregnancy and post-partum weights were evaluated according to WHO classification criteria for BMI [48]. The rate of pregnancy weight gain was assessed according to the Institute of Medicine BMI criteria for pregnant women [49]. Waist circumference will be classified according to the International Diabetes Federation’s cut-off for abdominal obesity in females of Middle Eastern origin (≥80 cm) [50]. Percent body fat will be measured by bioelectrical impedance (Tanita WB 100A digital scale); values >32% will be classified as high [51]. Blood pressure will be evaluated according to the National Heart, Lung, and Blood Institute of the National Institute of Health criteria [52]; values of systolic (SBP) >140 mmHg or diastolic blood pressure (DBP) >90 mmHg indicate hypertension.

**Gestational Diabetes Mellitus (GDM)**: GDM was diagnosed according to the National Institute for Health and Care Excellence (NICE) Diabetes in Pregnancy [53] guidelines. Blood glucose was measured and analyzed at the PHC/MCHs. All participants did a 2-hour 75 g Oral Glucose Tolerance Test (OGTT) to test for GDM. The latter was defined as a 2-hour plasma glucose level ≥7.8 mmol/L (140 mg/dl). The results were obtained from the mothers’ medical record.

**Infant anthropometric assessment**: measurements of head circumference, length, weight and mid-upper arm circumference (MUAC) will be performed following standard techniques by nurses at the hospitals, PHCs/MCHs. Weight and length measurements will be used to assess stunting, wasting, underweight, overweight, and obesity, following WHO criteria on child growth standards [54]. The MUAC will be used to assess the infant’s nutritional status, following WHO and UNICEF criteria [55]; values <11.5 cm indicate severe under-nutrition (wasting), 11.5–12.5 cm indicate moderate under-nutrition, and >12.5 cm indicate that the infant is well nourished.

### Biochemical assessments

**Blood profile**: maternal and infant biochemical assessments include fasting serum lipid profile (triglyceride, total cholesterol, high density lipoprotein (HDL) and low density lipoprotein (LDL)-cholesterol), glucose, insulin, high molecular weight (HMW) adiponectin, high-sensitivity C-reactive protein (*hs*-CRP), Tumor necrosis factor (TNF)-α and interleukin (IL)-6 and vitamin D. Following the delivery, maternal blood samples (10 mL) were collected by certified phlebotomists in appropriate test tubes, centrifuged and temporarily stored, within 30 minutes of collection, in hospital freezers (-80°C). Stored samples were transported monthly in an icebox from the hospitals to the University of Sharjah, Sharjah Institute for Medical Research (SIMR) Laboratories, for storage (-80°C) and analysis. During postpartum, at the 6^th^ month-visit, maternal (10 ml) and infant (5 mL) blood samples will be collected, processed and aliquoted in PHCs/MCHs’ laboratories and transported directly to the SIMR laboratories in an icebox for storage and analysis.

**Breast milk profile**: the assessment includes proximate analysis, fatty acid, oligosaccharides, HMW adiponectin, *hs*-CRP, TNF-α, IL-6 and insulin and vitamin D. Breast milk samples (10 mL) will be collected, upon delivery and during 6 months postpartum, using commercially available electrical breast pumps by the mother or the nurse, and placed in sterile polyproylene tubes, which ensures the collection of fore, mid, and hind milk within each sample. The samples will be directly transported to the SIMR laboratories for storage (-80°C) and analysis.

### Perinatal information

Perinatal information was obtained from the mother’s medical records at the hospital in which the delivery took place. These include occurrence of complications and GDM during pregnancy and delivery, delivery method, gestational age, date of delivery, sex, weight, length, and head circumference of infants.

### Quality assurance procedures

Several quality control measures are undertaken to ensure validity and reliability of collected data. Prior to data collection, collectors followed extensive training on study procedures, standardization of data collection and minimization of interviewer bias, and were provided with a comprehensive operation manual. This training was done in collaboration with experts from the Department of Nutrition and Food Sciences at the American University of Beirut (AUB). Manuals describing data collection procedures were developed and administered to participating centers. Data collection instruments were identical in all sites and were calibrated. Quality checks are routinely conducted for entered data by trained research assistants and one statistician. Finally, since birth cohorts often face planning and implementation challenges [56,57], the MISC research team sought to document encountered challenges and addresses them in a systematic manner. The challenges were noted through weekly meetings with the research assistants, and discussions with the participants and the nurses in the PHCs/MCHs, and responding strategies were planned based on researchers’ experiences and implemented after discussions within the research team, consultations with key scholars and support by relevant literature.

### Statistical analysis

Collected data are entered and analyzed using the Statistical Package for the Social Sciences (SPSS) software version 21.0. Six categories of variables are planned to be generated: maternal factors, pregnancy complications, birth outcomes, dietary intake, feeding practices and infant factors. The normality of data for all variables will be examined using Shapiro-Wilk test. For normally distributed continuous variables, means and standard deviations will be presented, and Student’s *t* test will be used. For skewed data, medians and interquartile ranges will be generated and Mann-Whitney *U* test will be performed. For categorical variables, frequencies will be presented, and Pearson Chi-square and Fisher’s Exact tests will be used, as appropriate. The associations between the variables will be examined using multiple linear regressions when the outcome of interest is continuous, and logistic regressions when the outcome considered is categorical. As a first step, the outcome variables will be cross-tabulated against potential confounders; those with *p*-values <0.2 will be entered into the model. A *p*-value <0.05 indicates statistical significance.

Finally, a comparative analysis is planned for baseline sociodemographic characteristics and exposures of participants who remained in the cohort and those who were lost to follow-up. If both groups are found to be similar, it is highly plausible that participants who finished the study and based on whom the conclusions will be generated are representative of the population who enrolled in the cohort. If significant differences are found with respect to main assessed factors, minimum requirements for addressing attrition will be reported [58] and alternative analytical approaches will be used to handle missing values of participants who were lost to follow-up [59].

## Results

### Participants’ characteristics

420 pregnant women in their third trimester were invited to participate, and 266 accepted to enroll (63.3%). As reasons for non-enrolment, our record showed that 36% (55/154) were not interested, 27% (42/154) declined due to the long duration of the cohort, and 20% (31/154) refused to enrol due to husband disapproval, while the remaining 17% (26/154) did not enrol because of blood collection. Ten mothers, who initially agreed to enroll, had to be excluded, since they had to leave before completing the baseline questionnaire due to time constraints. They did not differ in their characteristics from the other participants. Only 256 mothers completed the baseline questionnaire. Their baseline characteristics are shown in Tables 2 and 3. The mean age of the participants was 30.5±6.0 years, more than half of them were non-Emirati and the majority were university graduates and not employed. The majority of our starting sample was multiparous (76.6%) and up to 60% were either overweight or obese before they were pregnant. All except 7 were non-smokers (97.2%); however, 105 (40.5%) were subject to passive smoking. The prevalence of GDM was 19.2%. Between inclusion and delivery, 48 women were lost to follow-up, resulting in 208 women-infant pairs (dropout rate: 18.7%). The mean gestational age at delivery was 38.7±1.5weeks. One-third of the infants were delivered by caesarean section, and about half of the newborns were boys. Finally, 5.3% and 5.7% of pregnancies resulted in low birthweight and macrosomia, respectively (Table 4).

**Table 2:** Baseline sociodemographic characteristics of study participants.

Sociodemographic characteristics of pregnant women (n = 256)	N(%)
**Nationality**	
Arab Emirati	105(41.0)
Arab non-Emirati	151(59.0)
**Age of women (years)**	
<25	57(22.3)
25–29.9	62(24.2)
≥30	137(53.5)
**Education level**	
Intermediate or less	35(13.7)
High school/Technical Diploma	140(54.7)
University	81(31.6)
**Family monthly income (AED)**	
<5,000	18(7.0)
5,000–10,000	72(28.1)
>10,000	103(40.2)
Does not know/Refused to answer	63(24.6)
**Employment status**	
Housewife	211(82.4)
Employee	45(17.6)
**Maternity leave**	
1 month	14(5.5)
2 months	18(7.0)
3 months	9(3.5)
Does not know	4(1.5)

**Table 3:** Baseline clinical characteristics and practices during pregnancy of study participants.

**Maternal clinical characteristics (n = 256)**	**N(%) or Mean±SD**
**Parity**	
Primiparous	60(23.4)
Multiparous	196(76.6)
**Pre-gestational BMI (Kg/m**^**2**^**)**	
Underweight	11(4.3)
Normal Weight	93(36.3)
Overweight	76(29.7)
Obese	76(29.7)
**Blood pressure (mm Hg)**	
Systolic blood pressure	113.7±10.5
Diastolic blood pressure	66.1±8.5
**GDM**	
Yes	49(19.2)
No	207(80.8)
**Practices during pregnancy (n = 259)**	**N(%)**
**Smoking**	
Non-smoker	249(97.3)
Smoker	7(2.7)
**Smoking among family members**	
Husband	94(36.7)
Other family member	11(4.3)
**Physical activity (METs)**	
Low intensity activity	137(53.5)
Moderate intensity activity	59(23.1)
High intensity activity	31(12.1)
No physical activity	29(11.3)
**Vitamin/mineral supplements**	
Yes	218(85.2)
No	38(14.8)

BMI: body mass index; GDM: gestational diabetes mellitus; MET: metabolic equivalents

**Table 4:** Baseline delivery and infant characteristics.

**Delivery characteristics (n = 208)**^a^	**N(%) or Mean±SD**
**Complications during pregnancy**	
No	164(78.8)
Yes	44(21.2)
**Type of delivery**	
Normal	139(66.8)
Caesarean section	69(33.2)
**Gestational age at delivery (weeks)**	38.7±1.5
**Infant characteristics (n = 208)** ^a^	**N(%) or Mean±SD**
**Sex**	
Male	117(56.3)
Female	91(43.7)
**Birth head circumference (cm)**	34.2±1.3
**Birth length (cm)**	50.1±2.3
**Birth weight**	
Low weight (<2.5 Kg)	11(5.3)
Normal weight (2.5–4.0 Kg)	185(89.0)
Macrosomia (>4.0 Kg)	12(5.7)

^a^Date of running analysis: There were 208 delivered women out of 256 pregnant women

### Challenges and responding strategies

First, we underestimated the number of required staff and we faced a high turnover of trained research assistants that could not be easily replaced. This resulted in supplementary training sessions for newly recruited agents, time delays, and additional costs. Furthermore, data collection used interviewer-administered questionnaires, which is resource-intensive, in terms of human capital, cost and time. Second, the recruitment was limited by the participants’ concerns of study duration or lack of interest. Also, some mothers were reluctant to enroll before obtaining their husband’s approval. To develop and maintain confidence and familiarity between the participants and research team, we established a nurse-liaison in each participating center, where a key nurse acted as a facilitator between involved parties (research assistants, PHCs/MCHs administration, and participants). Third, data collection at delivery was extremely challenging. Same-day discharge and shortened post-delivery hospital stays offered limited time to collect information and samples from mothers and neonates at the hospital. I response, we adopted a daily tracking of participants approaching their due dates. Despite this, some women were missed at delivery or discharge. Participant retention was also critical. Common reasons for attrition were the inability to locate participants usually due to disconnected communication, lack of interest of some participants and husbands’ disapprovals. Several actions were taken to enhance retention, such as offering incentives (e.g. gift vouchers, infant toiletries…), obtaining other contact detail information for participants who consistently did not answer phone calls, and maintaining communication with the participants through phone calls or text messages. Moreover, we highlight the importance of discussing and negotiating the research details with the husbands in order to enhance their understanding of the study’s significance, and their approval to their wives’ participation.

Blood collection, especially from infants, was our biggest anticipated challenge. It was sometimes surrounded by technical difficulties and parents’ refusals. When needed, specialized pediatric nurses were hired to perform blood withdrawal. Finally, maintaining the quality of collected data is an ongoing challenge, requiring a rigorous information system, periodic data tracking and quality check, and unanimous adaptation of pre-specified data collection and management protocols (Table 5).

**Table 5:** The MISC: Challenges and responding strategies.

Challenges	Responding strategies
**Launching the cohort**	
Underestimating number of staff required	Ongoing hiring and training
High turnover of research assistants	Supplementary training (time/cost implications)
**Participant recruitment**	
Concerns of study duration	Establishing nurse-liaison (maintain confidence and familiarity between involved parties)
Lack of interest	
Reluctance to enroll before obtaining husband’s approval	
**Data collection**	
Notification of delivery often overlooked	Daily tracking of participants approaching due dates
Same-day discharge, shortened post-delivery stays (limited time to collect information and samples at the hospital)	Providing the mothers with necessary supplies and instructions on sample collection at home and arranging a pick-up the next day
Milk supply is not fully developed	
Difficulty to provide milk with a pump	
Blood collection (especially from infants)	Recruiting specialized pediatric nurses
Technical difficulties/Parental refusal	
Quality assurance	Rigorous information system infrastructure Periodic data tracking and quality check Unanimous adaptation of pre-specified data collection and management protocols
Data collection using interviewer-administered questionnaire (resource-intensive)	Relocate additional cost
**Participant retention**	
Participant fatigue	Maximize participant convenience: Efficiency in data collection (challenging with multi-component questionnaires) Flexibility of data collectors to commute between study sites at convenient times for participants
Missed appointments and late arrivals	Reminders through direct communication at each data collection time-point
Lack of interest	Maintaining communication (phone calls/messages) Offering incentives Involve the husbands in research details and raise their awareness
Husbands’ reluctance	
Repeatedly missed appointments	Obtaining other contact detail information Repeated efforts for restoring communication Rescheduling the infant vaccination appointment to increase convenience to the mothers (within acceptable dates for vaccinations) Exclusion
**Methodological considerations**	
Potential unequal distribution of confounders and inherent dissimilarities between-groups	Collecting data relating to numerous potential confounders Careful control through statistical analyses
Potential differential loss to follow-up	Comparative analysis of baseline and last-observation characteristics between attired/retained participants
Limited ability to generalize the results to non-Arab residents	Priority of obtaining ethnic-specific data due to current lack of evidence

## Discussion

This article described the details of the methodology and the challenges as well the baseline characteristics of the participants in the MISC cohort study. The MISC is the first effort in the UAE, and among the very first research projects in the Arab world, to prospectively address key questions related to women and children’s health. Being a longitudinal study, the MISC runs the advantage of providing descriptive, as well as etiological information. Accordingly, it is anticipated to generate results that overcome available evidence in the UAE, which is mostly focused on adults and limited by its cross-sectional nature.

Existing and planned birth cohort studies are urged to encourage implementation experience and data sharing to leverage the initial investment required to establish these cohorts and enable cross-comparisons. This contributes to the efforts aiming to advance scientific knowledge and practice from a local to global levels. The MISC joins regional and international birth cohort studies endeavors investigating early metabolic programming of lifelong health. Specifically, a comparison of country-specific results within the Arab region is planned with the Mother and Infant Nutritional Assessment (MINA) cohort study [25]. Moreover, the MISC will collaborate with ongoing international birth cohorts through networking, data sharing and pooling, by being part of the Birth Cohort Consortium of Asia (BiCCA) (http://www.bicca.org/).

Our findings are expected to critically inform the design of sustainable, effective and cost-effective interventions aiming to promote infant health. The MISC goes hand in hand with the governmental vision and national efforts aiming to promote and enforce the implementation of international guidelines on infant and young children feeding in the UAE, in particular the WHO and UNICEF Baby Friendly Hospital initiative (WHO/UNICEF BFHI) [60] and Infant and Young Child Feeding (IYCF) [61]. Within this scope, the findings of the project are also much needed to advocate for the implementation of supportive policies for women during lactation and early childhood, such as adequate maternity leave, breastfeeding breaks and flexibility. Finally, the MISC intends to generate information that could eventually be used to curb the NCD epidemic in future generations, thus reduce pertaining preventable morbi-mortality burden. This is in line with global [62], regional [31], and national [32] agendas.

The preliminary results reveal that our sample is skewed towards housewives, multiparous, well educated, from economically well-off households, and vaginally delivering women. All of which are documented predictors of the duration and exclusivity of breastfeeding in the UAE [30]. Furthermore, our preliminary results showed that 29.7% were overweight and 29.7% were obese before pregnancy. These percentages are below previous estimates among adult Emirati females (overweight: 31.4% and obesity: 34.2%) [27]. It is arduous to compare our sample with those of the general population of Arab women of childbearing age and their offspring in the country, mostly due to the lack of pertaining published data. The prevalence of GDM among participants (18.9%) falls within global estimates (<1%-28%) [63], and within those reported from the UAE (7.9%-24.9%) [64]. This prevalence is however different than rates reported from other countries, i.e. 24% in Saudi Arabia [65] and 16.3% in Qatar [66]. Benchmarking against other studies is challenging, taking the diversity of definitions used to diagnose GDM [67]. While we acknowledge these limitations, we do not expect them to hinder the interpretation of our data, since we aim in this cohort to longitudinally investigate determinants of infant health and early-life cues of NCDs, rather than generating national prevalence of breastfeeding, NCDs or their risk factors.

### Strength and limitations

The MISC is a longitudinal study integrated within the healthcare system, rather than operating in the idealized experimental environment, consequently allowing the generation of “real life” data [68] in an ethical and practical fashion. The study entails comprehensive and systematic prospective quantitative assessments of mothers and children. It provides an all-inclusive analysis of the determinants of pregnancy, birth, mothers and children’s hard and proxy outcomes, without recurring to participant recall, and allows the suggestion of a clear temporal sequence between exposures and outcomes. Another strength of this study is the adoption of culturally-adapted data collection tools. Furthermore, the interviewer-administered questionnaires used in this study are expected to provide consistency and clarity in the administration and understanding of questions, unlike self- administered questionnaires.

The MISC aims to explore new perspectives in “fetal programming” and management of NCDs. Specifically, the MISC is first to explore the effect of early-life exposures on the cognitive development of young Arab children and is expected to enrich existing international evidence [69–71]. In addition, the MISC addresses numerous novel biomarkers, and pioneers in exploring these biomarkers specifically in children, by correlating maternal milk and blood biomarkers with infant blood biomarkers and immediate outcomes, in people with Arab ethnicity. Accordingly, results from the MISC complement the international evidence on the association between maternal and infant biomarkers and their effects on infant physiology [72–74]. In particular, the MISC will evaluate the specific short-term effects of lactation on maternal body composition (adiposity), as well as maternal and child cardio-metabolic homeostasis, and anti- and pro-inflammatory biomarkers. If lactation is found to exert beneficial metabolic and cognitive effects, then the translation of the findings from the MISC may have a significant public health impact. Lactation promotion is expected to be a low-cost, feasible strategy for the prevention of NCDs and promotion of intergenerational health [75,76]. The findings of MISC may be applicable to other country members of the Gulf Cooperation Council, exhibiting similar cultural, social and economic characteristics, and are similarly struggling to contain and mitigate the NCDs epidemic. Finally, the MISC design and measurements are consistent with those of other regional and international birth cohorts. This is expected to facilitate comparisons across-studies and improve the power to identify predictors of health and disease among infants.

As for the limitations, first, since mothers were not randomly assigned to different exposure groups, unequal distribution of confounders between-groups of comparison, and inherent dissimilarities relating to the mothers’ choice of specific behaviors might lead for possible biases. Therefore, we collected data relating to numerous potential confounders, and we planned to control for them through statistical analyses. This shall minimize bias from reverse causation or unmeasured confounding and might provide a robust alternative to the randomization of mothers which is unlikely to be feasible or ethical. Second, we could not collect information about non-enrollees because they did not consent to participate in the study. It would have been interesting to compare their characteristics with the participants to identify potential biases in recruitment. However, we do not expect to have major differences regarding their characteristics that could influence our results. Future cohorts could benefit from our experience in this regard, and not oversee this limitation. Furthermore, differential loss to follow-up could introduce bias. Subsequently, we planned for a comparative analysis of baseline and last-observation characteristics between women (and their children, where applicable) who attrited and those who completed the cohort. Finally, the exclusion of non-Arab women limits our ability to generalize our results to the population residing in the UAE. However, we only included Arab women to avoid any ethnic variability in the evaluation of adiponectin, as well as its relationship with other biomarkers [77–80]. We prioritized this issue over the possibility of generalizing our findings, taken the lack of available evidence related to adiponectin.

Finally, the implementation of this cohort was subject to many logistical, methodological and sociocultural challenges that required tailored remedial strategies. Many of these challenges are common to birth cohorts [56,81,82]. At this stage, we recommend that the complex ethical issues involved in conducting a birth cohort, must be carefully considered. Moreover, the necessary start-up time, staff, and costs must not be underestimated and funding of extra costs relating to logistical complexity and added expenses must be accounted for. Moreover, it is recommended that future cohort studies actively include the father as a participant, and examine the paternal factors that contribute to NCDs in infants. This is suggested to strengthen the study scientifically and logistically.

## Conclusion

The present article details the design and methodology of one of the first birth cohorts in the Arab region. It is also the first documentation of practical strategies used to conduct a birth cohort in the UAE. Since “new birth cohort studies are a resource for the present as well as the future” [83], the MISC is intended to enhance our understanding of predictors of infant health and early-life cues of NCDs. It is also intended to be the backbone for the development of country-specific nutritional guidelines during gestation and childhood, and a basis for the implementation of public health initiatives aiming to promote maternal and infant health and curb the NCDs epidemic. The MISC attempts to be a model for future cohort studies in the region in terms of design, conduct, reporting and public health advocacy.
